# Clinical Whole-Body Gait Characterization Using a Single RGB-D Sensor

**DOI:** 10.3390/s25020333

**Published:** 2025-01-08

**Authors:** Lukas Boborzi, Johannes Bertram, Roman Schniepp, Julian Decker, Max Wuehr

**Affiliations:** 1 German Center for Vertigo and Balance Disorders (DSGZ), LMU University Hospital, LMU Munich, 81377 Munich, Germany; 2 Institut für Notfallmedizin und Medizinmanagement (INM), LMU University Hospital, LMU Munich, 80336 Munich, Germany; 3 Schön Klinik Bad Aibling, 83043 Bad Aibling, Germany; 4 Department of Neurology, LMU University Hospital, LMU Munich, 81377 Munich, Germany

**Keywords:** gait analysis, gait disorders, motion tracking, pose tracking, RGB-D sensor

## Abstract

Instrumented gait analysis is widely used in clinical settings for the early detection of neurological disorders, monitoring disease progression, and evaluating fall risk. However, the gold-standard marker-based 3D motion analysis is limited by high time and personnel demands. Advances in computer vision now enable markerless whole-body tracking with high accuracy. Here, we present vGait, a comprehensive 3D gait assessment method using a single RGB-D sensor and state-of-the-art pose-tracking algorithms. vGait was validated in healthy participants during frontal- and sagittal-perspective walking. Performance was comparable across perspectives, with vGait achieving high accuracy in detecting initial and final foot contacts (F1 scores > 95%) and reliably quantifying spatiotemporal gait parameters (e.g., stride time, stride length) and whole-body coordination metrics (e.g., arm swing and knee angle ROM) at different levels of granularity (mean, step-to-step variability, side asymmetry). The flexibility, accuracy, and minimal resource requirements of vGait make it a valuable tool for clinical and non-clinical applications, including outpatient clinics, medical practices, nursing homes, and community settings. By enabling efficient and scalable gait assessment, vGait has the potential to enhance diagnostic and therapeutic workflows and improve access to clinical mobility monitoring.

## 1. Introduction

Gait disturbances are among the most common symptoms in neuro-geriatric patients. Characterizing gait alterations is crucial in clinical practice for the early detection of neurodegenerative diseases, monitoring disease progression or the effects of interventions, and assessing individual risk profiles such as the propensity to fall. The clinical and neurobiological complexity and heterogeneity of common gait disorders necessitate precise and granular phenotyping [1,2,3]. This is typically achieved using easily applicable disease-specific scores, such as the Unified Parkinson’s Disease Rating Scale (UPDRS [4]), the Expanded Disability Status Scale (EDSS [5]), and the Scale for the Assessment and Rating of Ataxia (SARA [6]). However, these scores are limited by factors such as low sensitivity and examiner dependency, leading to poor reproducibility and an inability to detect subtle prodromal signs of neurodegenerative diseases [7,8,9].

These limitations can be resolved through instrumented gait analysis, which is increasingly used in clinical settings to enable an unbiased, high-resolution phenotyping, monitoring, and risk stratification of spatiotemporal gait alterations. Walking is a complex process involving the entire body, extending beyond mere spatiotemporal step placement. Kinematic pathosignatures of neurological gait disorders frequently manifest at different sites of the body—for example, disturbed interlimb coordination in ataxic gait [10] or altered trunk bending and arm swing coordination in hypokinetic gait [11]. The gold standard for instrumented assessment of whole-body kinematics during walking is marker-based 3D motion capture systems, which accurately record spatiotemporal gait kinematics using synchronized cameras from multiple perspectives. However, these systems are expensive, space- and resource-intensive, and require specially equipped laboratories and significant personnel and time; they are, therefore, seldom used in routine clinical assessments.

Advances in computer vision have made reliable 2D pose tracking without body markers possible [12]. Building on this innovation, markerless multi-camera systems can now achieve accuracy levels for 3D pose tracking comparable to the gold standard [13,14,15]. Nonetheless, they remain equipment-intensive and require substantial space. Combined color and depth cameras (RGB-D) have also been successfully used in the past [16,17,18,19,20], but to obtain accurate spatiotemporal representations of gait over longer distances, multiple cameras had to be connected to compensate for limited depth coverage and noise in depth mapping [21,22,23].

Recently, new generations of RGB-D sensors have emerged, offering greater depth coverage (up to 5 m) and improved accuracy in depth mapping. In this study, we explore the potential of using a single RGB-D sensor in combination with a state-of-the-art pose-tracking algorithm for comprehensive 3D whole-body visual gait assessment (vGait). Analyzing gait with a single sensor would be widely and flexibly applicable due to its resource and space efficiency. We validated the vGait method in a cohort of participants, focusing on its ability to represent gait when walking toward the camera (frontal perspective) and when walking laterally across the camera’s view (sagittal perspective). Our evaluation considered a comprehensive selection of established gait parameters that capture the spatiotemporal step sequence, aspects of whole-body coordination during walking, and clinically relevant granular gait changes, particularly step-to-step fluctuations and asymmetries in gait patterns. Our findings have the potential to advance the adoption of gait analysis in clinical practice by providing a cost-effective and spatially flexible tool for the early detection and monitoring of gait disturbances across diverse patient populations.

## 2. Materials and Methods

### 2.1. Participants

Fifteen healthy individuals aged between 22 and 62 years (mean age: 32.5 ± 11.8 years; height: 1.78 ± 0.09 m; weight: 77.9 ± 14.1 kg; 5 females) participated in the study. All participants provided written informed consent prior to inclusion and were screened for any neurological or orthopedic conditions that could affect balance or locomotion.

### 2.2. Experimental Procedures

**Figure 1:**
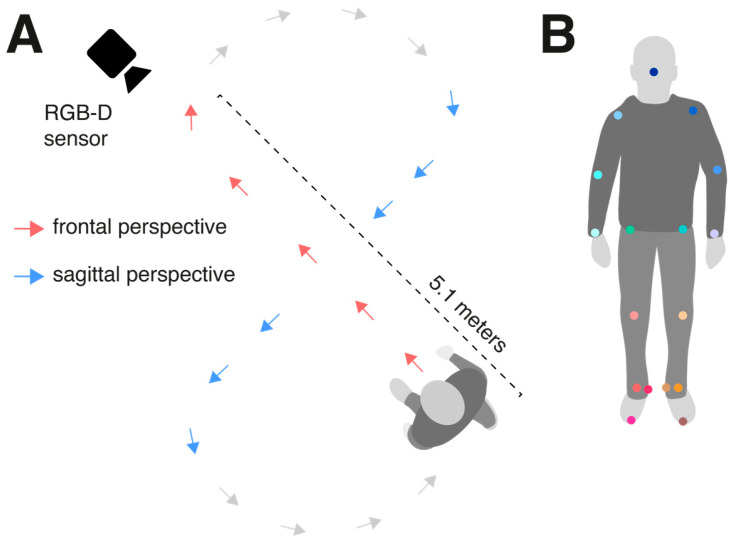
Experimental setup. (**A**) Participants walked along a marked figure-eight path with a diagonal length of 5.1 m, allowing for both frontal-perspective and sagittal-perspective walking. (**B**) A total of 17 displayed keypoints were analyzed to calculate spatiotemporal gait cycle parameters.

The gait behavior of each participant was recorded at a self-selected walking speed. To obtain continuous and extended recordings of the gait sequence from different perspectives (i.e., frontal and sagittal) that simulate various spatial application scenarios for gait assessment, participants walked along a marked figure-eight path on the floor with a diagonal length of 5.1 m (see Figure 1A). The entire gait sequence lasted 3 min.

The evaluated vGait method utilized an RGB-D sensor (Azure Kinect, Microsoft, Redmond, WA, USA) mounted on a tripod at a height of 1.4 m (see Figure 1A). Raw data (including RGB images at a resolution of 3840 × 2160 pixels and depth images at a resolution of 640 × 576 pixels with a depth field of view (FOV) of 75° × 65°) were captured at a fixed sampling rate of 30 Hz using the Kinect Azure Sensor SDK. The sensor was positioned such that participants walked along a straight path toward the sensor (frontal perspective) and along a straight path laterally across the camera’s view (sagittal perspective).

The Azure Kinect sensor comes with its own pose-tracking SDK provided by Microsoft. In a previous study [24], we observed during two simple gait tasks (walking in place and a short walking distance toward the sensor) that this pose-tracking module had significant weaknesses, particularly in capturing ankle and foot movements. As a result, even basic gait parameters such as cadence, speed, and average step length showed only poor to moderate agreement with the gold standard. For this study, we therefore used a freely available, more accurate pose-tracking approach. The markerless 3D pose estimation was conducted in a two-step process. Initially, a 2D pose estimation was performed on the RGB stream of the sensor using a top-down approach. First, the participant was identified using a bounding box detector (YOLOv8 [25]). Then, within the bounding box, the full-body pose, comprising 26 body and foot keypoints from the COCO WholeBody annotations, was predicted using RTMPose [26]. Finally, the 2D keypoints were projected into 3D coordinates using the depth stream of the sensor. For the full-body gait analysis, 17 keypoints were selected (see Figure 1B).

To validate vGait, we employed a marker-based multi-camera motion capture system as the clinical reference standard (Qualisys AB, Gothenburg, Sweden). The system consisted of nine wall-mounted cameras that captured full-body motion within a square area of approximately 3.6 m × 3.6 m—the diagonal of this area (5.1 m) overlapped with the diagonal length of the figure-eight walking path. The motion capture system was configured to record at 178 Hz. Prior to measurements, extensive spatial calibration of the system was performed. Lightweight passive infrared (IR) reflective markers with a diameter of 19 mm were placed on 36 predefined anatomical landmarks to capture the motion of all major body joints, including the head and trunk. The markers were affixed to the participants’ tight-fitting clothing or directly onto bare skin to ensure accurate motion tracking.

### 2.3. Data Analysis

First, the keypoint trajectories from the Qualisys recordings were down-sampled to 30 Hz to match the sampling rate of the Azure Kinect recordings. Then, the motion trajectories from both recordings were smoothed using a 4th-order Butterworth low-pass filter with a cutoff frequency of 7 Hz, and small gaps (<250 ms) in the data were filled using cubic spline interpolation. The subsequent analysis for step detection and gait parameter evaluation was restricted to the sections where both systems could simultaneously observe the walking participant, which corresponded to the two diagonal segments of the figure-eight path. The analysis was conducted separately for gait segments in the frontal and sagittal planes.

Step detection was performed using an established gait event detection algorithm based on an adaptive threshold method utilizing foot keypoints [27]. Candidate initial ground contact (IC) time instances were identified as those where the magnitude of the 3D heel velocity vector became less than 0.5 times the walking speed. For the detection of final ground contact (FC) time instances, an adaptive threshold of 0.8 times the walking speed was applied to the 3D toe velocity. The detection algorithm was executed twice: initially with an estimated walking speed of 1 m/s and subsequently with a refined walking speed estimate based on the IC times and positions identified in the first iteration.

**Figure 2:**
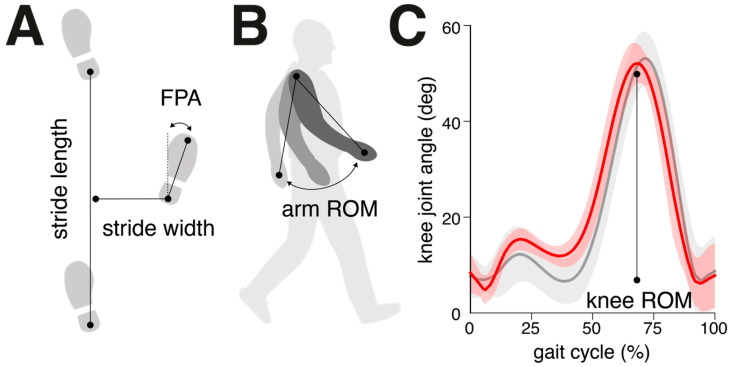
Definition of spatial gait characteristics. (**A**) Stride length is the distance between two successive heel contacts of the same foot, while stride width is the perpendicular distance from one heel contact to the line connecting two successive heel contacts of the opposite foot (i.e., the line of progression). The FPA is the angular deviation between the foot midline and the line of progression. (**B**) Arm swing ROM is the maximal angular displacement of the line connecting the shoulder and wrist in the walking direction within a gait cycle. (**C**) Knee ROM is defined as the angular difference between the maximum extension and flexion of the knee during the gait cycle. Exemplary knee joint angle curves (mean ± SD) are shown from vGait (red line) and the ground truth (gray line). Abbreviations: FPA, foot progression angle; ROM, range of motion.

Based on the results of the step detection, various gait cycle parameters were calculated to characterize the spatiotemporal step sequence and different additional aspects of whole-body coordination during walking (Figure 2). The spatiotemporal gait cycle parameters included stride time (temporal difference between successive ICs of the same foot, [s]), swing phase (duration of the gait cycle during which only one foot is in contact with the ground, [s]), and double support phase (duration of the gait cycle during which both feet are in contact with the ground, [s]). Additionally, stride length (Euclidean distance between the foot positions at successive ICs of the same foot, [m]) and stride width (perpendicular distance of one IC foot position to the line connecting two successive IC positions of the opposite foot, [m]). Furthermore, we calculated the foot progression angle (FPA, angle between the line of progression, i.e., the line connecting two successive IC positions of the same foot, and the longitudinal axis of the foot, defined by the line connecting the heel and toe positions during the stance phase, [°]). The arm swing range of motion (ROM) was defined as the maximal angular displacement of the line connecting the shoulder and wrist in the walking direction during the gait cycle [°]. The knee ROM was determined by the angular difference between the maximum extension and flexion of the knee during the gait cycle [°]. For all gait parameters, we calculated the mean over all collected gait cycles. Furthermore, stride-to-stride fluctuations in each parameter were assessed by calculating the coefficient of variation (CV; 100 × std/mean, [%]), and side asymmetry was computed using the formula 100 × (1mean(smaller foot value)/mean(larger foot value) [%].

### 2.4. Statistical Analysis

The performance of vGait was evaluated with respect to (1) the detection performance and temporal agreement of identified gait events, i.e., ICs and FCs with the gold-standard method, and (2) the agreement of derived spatiotemporal gait metrics with the gold standard.

The overall detection performance measured the number of events detected by vGait with corresponding gold-standard events (true positives, *TP*), the number of gold-standard-detected events missed by vGait (false negatives, *FN*), and the number of vGait-detected events that were not detected by the gold standard (false positives, *FP*). Using these metrics, detection performance was primarily evaluated by the *F*1-*score*, which calculates the harmonic mean of *precision* and *recall*. The *F*1-*score* ranges between 0 and 1, reflecting the worst and best performance, respectively:

$$recall=\frac{TP}{TP+FN}$$

$$precision=\frac{TP}{TP+FP}$$

$$F1\;score=2\times \frac{precision*recall}{precision+recall}$$

A detected event (either IC or FC) was considered a *TP* if the absolute time difference from the corresponding gold-standard event was <250 ms [28]. For all *TP*, the time agreement with the ground truth was quantified by $temporal\;error=abs(t_{gold\;standard}-t_{vGait})$.

We employed multiple statistical techniques to assess the agreement of derived temporal and spatial gait cycle parameters with the gold standard, including the absolute and relative root mean square error (RMSE) and the intraclass correlation coefficient for relative agreement (ICC(3,1); two-way mixed model). ICC outcomes were interpreted according to established categories [29]: poor agreement (<0.5), moderate agreement (0.5–0.75), good agreement (0.75–0.9), and excellent agreement (>0.9). All analyses were conducted using Python 3.9.

## 3. Results

### 3.1. Step Detection Performance

**Figure 3:**
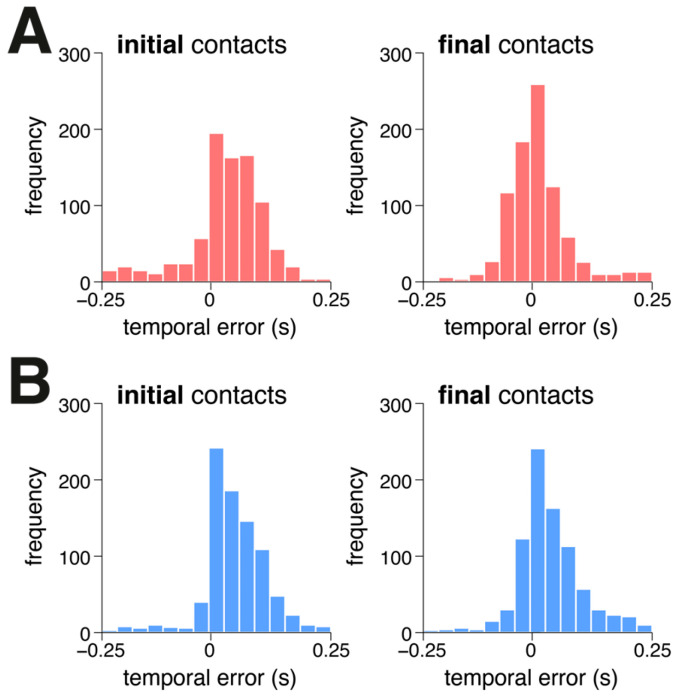
Histograms illustrating the temporal agreement (t_gold standard_–t_vGait_) of initial and final foot contacts identified by vGait compared to the gold standard during (**A**) frontal-perspective walking and (**B**) sagittal-perspective walking.

Table 1 and Figure 3 summarize the overall performance of vGait in detecting ICs and FCs during frontal- and sagittal-perspective walking. Both event types were identified with high accuracy, achieving F1 scores exceeding 95% for walking captured from both perspectives. Overall, vGait tended to detect both types of events slightly earlier, with absolute time errors ranging from 45 to 65 ms.

### 3.2. Accuracy of Gait Cycle Parameters

Using the detected temporal gait events, various spatiotemporal gait cycle parameters were calculated to characterize the stepping sequence (e.g., stride time, swing time, double support time, stride length, base of support, FPA) and aspects of whole-body coordination during walking (e.g., arm swing ROM, knee angle ROM). For each parameter, the mean, variability (CV), and side asymmetry were computed. An overview of the agreement between vGait-derived gait cycle parameters and the gold standard is provided in Table 2 for walking captured from the frontal perspective and in Table 3 for walking captured from the sagittal perspective. For frontal-perspective walking, all mean gait cycle parameters demonstrated good-to-excellent agreement. While some exceptions were observed (e.g., variability and side asymmetry of swing time and arm swing ROM), overall good agreement was also achieved for variability and side asymmetry metrics.

Agreement for sagittal-perspective walking was, in general, comparable to frontal-perspective walking, albeit with slightly reduced performance, particularly for variability and asymmetry estimates of gait cycle parameters.

## 4. Discussion

In this study, we investigated the reliability of a clinical gait analysis approach using a single integrated RGB-D sensor (vGait). Our findings demonstrate that this method enables reliable temporal step detection, as well as the determination of spatiotemporal gait parameters across various levels of granularity (i.e., mean values, variability, and side asymmetry) and clinically relevant aspects of whole-body gait coordination, with overall good-to-excellent reliability. This holds true when analyzing gait in a frontal perspective (i.e., walking in the direction of the camera) and, with only minor compromises, from a sagittal perspective (i.e., walking sideways across the camera’s field of view), thus underscoring the spatial flexibility of the vGait approach.

It is now common practice to categorize neurological gait disorders based on their phenotypic presentations (e.g., ataxic, hypokinetic, dyskinetic) [1,2,3], and optical solutions are ideally suited to instrumentally characterize these conditions. Although conventional marker-based motion capture systems have traditionally represented the gold standard, they remain time- and resource-intensive. Recent advances in computer vision and optical hardware have enabled more comprehensive “deep phenotyping” of gait disorders without markers or complex multi-camera setups. Indeed, a growing body of research now shows that state-of-the-art pose-tracking solutions can achieve accuracy levels approaching those of marker-based systems [13,14,15]. However, achieving such accuracy in 3D gait analysis has often required multi-camera RGB configurations with extensive calibration and synchronization, limiting their practical utility. Previous efforts to enhance spatial flexibility through integrated RGB-D sensors were hampered by insufficient depth coverage, excessive noise, and tracking errors, necessitating multiple sensors to reliably cover clinically meaningful distances [16,17,18,19,20,21,22,23]. With the latest generation of RGB-D sensors offering extended depth coverage and improved depth mapping, our current study demonstrates that these constraints have now been overcome, enabling clinically reliable gait phenotyping using a single, integrated, and spatially versatile sensor system.

Building on these advances, our study demonstrates that clinically reliable gait phenotyping is now achievable over meaningful distances using just a single RGB-D sensor (Kinect Azure). In the sagittal plane, we successfully characterized gait across ~5 m, capturing approximately eight steps or seven complete gait cycles, assuming an average step length of ~0.6 m. From a frontal perspective, we achieved similarly robust results over ~4.6 m, amounting to about seven steps or six full cycles. This spatial flexibility extends the applicability of vGait beyond specialized laboratory setups, making it readily deployable in a variety of clinical and non-clinical environments, such as outpatient clinics, medical practices, nursing homes, and community centers. Moreover, our approach is not confined to gait assessment alone; it can be seamlessly adapted to evaluate other clinically relevant parameters, including static and dynamic postural stability [24,30], thereby enriching clinical evaluations with objective, digital outcome metrics.

Clinical gait assessment commonly focuses on five major domains [31,32]: (1) pace (e.g., gait speed, stride length), (2) rhythm (e.g., swing and double support phases), (3) variability (e.g., stride time and stride length variability), (4) asymmetry (e.g., asymmetry of stride time and stride length), and (5) postural control (e.g., average and variability of stride width). Our results show that vGait can reliably quantify spatiotemporal parameters across all these domains, underscoring its potential for the comprehensive and accurate identification of gait disturbances. This level of precision enables clinicians to monitor disease progression and therapeutic responses with clinically meaningful accuracy. For example, the minimal clinically important difference (MCID) for gait speed in adults with various health conditions, such as multiple sclerosis, acute cardiovascular disease, and stroke, typically ranges from 10 to 20 cm/s [33,34]. This range is well above vGait’s error in estimating gait velocity (RMSE_ABS_ of about 4 cm/s). Beyond gait speed, changes in gait variability offer critical insights into fall risk and disease progression in conditions such as cerebellar gait ataxia and Parkinson’s disease [35,36]. Recent estimates suggest an MCID of 1.0% for spatial gait variability in patients with Parkinson’s disease [37], which aligns closely with vGait’s error in estimating stride length variability (RMSE_ABS_ = 1.5%). Similarly, the MCID for gait asymmetry—a key metric for assessing rehabilitation outcomes in stroke patients—has been estimated to range between 10 and 20% [38]. vGait meets these precision requirements with its stride time asymmetry error (RMSE_ABS_ of about 6%) and stride length asymmetry error (RMSE_ABS_ of about 2–3%).

Some limitations of vGait should be acknowledged. Thus far, we have only validated the approach in healthy participants. Nevertheless, since the underlying pose-tracking method identifies keypoints frame-by-frame and does not rely on a specific movement profile, we anticipate that its reliability will extend to pathological gait patterns [12]. Another limiting factor is the low temporal sampling rate of the RGB-D sensor used in this study (Kinect Azure), which operates at 30 Hz. Combined with the inherent temporal imprecision of the step detection algorithm (for both the gold standard and vGait) of >20 ms [27], these factors likely contribute significantly to the observed temporal variability and side asymmetry. This may explain why these aspects did not achieve excellent agreement with the gold standard. Future studies should aim to use RGB-D sensors with higher temporal resolution when possible. Finally, we validated vGait using a particular RGB-D technology (time-of-flight via Kinect Azure), so future studies should investigate whether other RGB-D systems—such as those integrated into smartphones or tablets, employing different depth-mapping technologies (e.g., stereo vision, structured light, LiDAR)—perform similarly. Such findings could pave the way for broadly accessible, cost-effective, and mobile 3D gait-analysis solutions.

## 5. Conclusions

In summary, we have demonstrated that clinically reliable gait analysis can be achieved using a single integrated RGB-D sensor (vGait), providing good-to-excellent agreement with gold-standard marker-based methods across a broad range of spatiotemporal gait parameters. Moreover, the approach can be flexibly applied from different perspectives. Future research should validate vGait in different patient populations, including individuals with hypokinetic or ataxic gait disorders, and explore its applicability to alternative RGB-D sensor technologies without significant loss in quality.

## Figures and Tables

**Table 1:** Detection performance and temporal agreement of initial and final foot contacts identified by vGait compared to the gold standard.

Perspective	Event Type	TP	FN	FP	Recall	Precision	F1	Abs. Time Error
frontal	initial contact	864	54	25	0.972	0.941	0.956	0.046 s
	final contact	866	48	10	0.989	0.947	0.968	0.063 s
sagittal	initial contact	843	38	18	0.979	0.957	0.968	0.051 s
	final contact	852	28	7	0.992	0.968	0.980	0.056 s

Abbreviations: TP: true positives; FN: false negatives; FP: false positives; F1: F1 score; abs: absolute.

**Table 2:** Accuracy statistics of gait cycle parameters derived from vGait during frontal perspective walking, compared to the gold standard.

Param.	Metric	vGait	Gold Standard	RMSE_ABS_	RMSE_REL_	ICC(3,1)
stride time	mean	1.1 ± 0.1 s	1.1 ± 0.1 s	0.1 s	4.6%	0.952
	CV	3.9 ± 0.6%	6.3 ± 3.1%	3.9%	61.3%	0.812
	asym.	1.6 ± 1.0%	5.3 ± 5.3%	6.4%	120.8%	0.803
swing time	mean	0.4 ± 0.0 s	0.5 ± 0.0 s	0.1 s	74.4%	0.784
	CV	9.2 ± 2.0%	2.4 ± 1.5%	7.3%	75.5%	0.579
	asym.	5.7 ± 4.5%	2.1 ± 2.1%	6.4%	110.0%	0.442
dsupp time	mean	0.2 ± 0.0 s	0.1 ± 0.1 s	0.1 s	74.4%	0.769
	CV	21.4 ± 4.9%	52.7 ± 23.6%	39.7%	75.5%	0.784
	asym.	8.5 ± 7.8%	30.9 ± 24.1%	34.0%	110.0%	0.778
stride length	mean	1.4 ± 0.1 m	1.4 ± 0.1 m	0.0 m	3.0%	0.986
	CV	3.4 ± 0.5%	5.1 ± 2.0%	2.9%	56.4%	0.738
	asym.	1.5 ± 0.8%	3.5 ± 3.5%	4.2%	188.5%	0.783
base of support	mean	0.2 ± 0.0 m	0.2 ± 0.0 m	0.0 s	11.5%	0.896
	CV	27.7 ± 9.9%	26.2 ± 8.3%	5.5%	53.5%	0.850
	asym.	10.5 ± 8.4%	13.7 ± 9.7%	5.8%	2.6%	0.859
velocity	mean	1.2 ± 0.1 m/s	1.2 ± 0.1 m/s	0.0 m/s	2.9%	0.991
	CV	5.3 ± 0.6%	3.5 ± 0.9%	2.0%	58.6%	0.787
	asym.	2.1 ± 1.9%	1.9 ± 2.1%	2.2%	112.8%	0.795
FPA	mean	7.5 ± 1.5°	5.9 ± 1.6°	2.0°	33.4%	0.912
	CV	25.8 ± 6.9%	28.2 ± 8.8%	9.1%	33.4%	0.805
	asym.	12.5 ± 9.0%	20.2 ± 15.5%	19.2%	95.5%	0.754
arm swing ROM	mean	31.1 ± 8.8°	25.2 ± 9.7°	7.5°	29.8%	0.949
	CV	29.6 ± 14.7%	17.5 ± 5.8%	17.9%	102.3%	0.522
	asym.	24.8 ± 13.6%	16.2 ± 13.2%	21.3%	131.4%	0.655
knee angle ROM	mean	38.2 ± 4.3°	40.3 ± 4.4°	4.8 s	11.9%	0.817
	CV	10.9 ± 3.5%	6.5 ± 5.0%	5.5%	102.0%	0.806
	asym.	5.6 ± 4.1%	7.8 ± 6.6%	5.8%	107.1%	0.730

Abbreviations: dsupp time: double support time; FPA: foot progression angle; ROM: range of motion; CV: coefficient of variation; asym.: asymmetry; RMSE: absolute and relative root mean square error; ICC: intraclass correlation coefficient.

**Table 3:** Accuracy statistics of gait cycle parameters derived from vGait during sagittal-perspective walking, compared to the gold standard.

Param.	Metric	vGait	Gold Standard	RMSE_ABS_	RMSE_REL_	ICC(3,1)
stride time	mean	1.1 ± 0.1 s	1.1 ± 0.1 s	0.0 s	3.75 %	0.975
	CV	4.4 ± 1.5%	1.5 ± 1.2%	3.4%	222.2%	0.638
	asym.	5.0 ± 3.4%	0.7 ± 0.9%	5.5%	758.1%	0.335
swing time	mean	0.4 ± 0.0 s	0.5 ± 0.0 s	0.1 s	13.6%	0.663
	CV	10.2 ± 3.4%	2.9 ± 3.0%	8.4%	292.8%	0.689
	asym.	8.7 ± 6.1%	2.6 ± 2.5%	9.6%	368.8%	0.365
dsupp time	mean	0.2 ± 0.1 s	0.1 ± 0.0 s	0.1 s	96.9%	0.741
	CV	30.7 ± 8.5%	18.9 ± 16.7%	20.9%	110.5%	0.790
	asym.	16.5 ± 8.1%	7.6 ± 6.7%	13.0%	172.0%	0.684
stride length	mean	1.4 ± 0.1 m	1.4 ± 0.1 m	0.0 m	2.1%	0.996
	CV	3.0 ± 1.1%	1.9 ± 0.8%	1.5%	77.3%	0.743
	asym.	2.1 ± 1.4%	0.7 ± 0.7%	2.2%	334.6%	0.473
base of support	mean	0.2 ± 0.1 m	0.2 ± 0.1 m	0.0 m	23.7%	0.959
	CV	28.7 ± 14.0%	28.9 ± 18.5%	12.0%	41.4%	0.908
	asym.	17.2 ± 10.2%	13.5 ± 9.2%	13.9%	102.5%	0.665
velocity	mean	1.3 ± 0.1 m/s	1.3 ± 0.1 m/s	0.0 m/s	2.9%	0.988
	CV	5.3 ± 2.2%	2.5 ± 1.6%	3.6%	144.8%	0.695
	asym.	4.1 ± 3.5%	0.6 ± 1.0%	4.6%	718.2%	0.451
FPA	mean	5.6 ± 2.1°	5.4 ± 2.1°	0.6°	10.4%	0.985
	CV	30.8 ± 14.0%	27.6 ± 14.7%	9.9%	35.8%	0.913
	asym.	34.2 ± 11.0%	19.5 ± 14.7%	15.2%	78.0%	0.810
arm swing ROM	mean	34.2 ± 11.0°	25.3 ± 9.5°	12.4°	49.3%	0.838
	CV	34.0 ± 8.3%	14.4 ± 8.5%	22.4%	156.3%	0.716
	asym.	36.2 ± 16.7%	17.9 ± 12.1%	29.3%	163.9%	0.545
knee angle ROM	mean	49.1 ± 6.9°	38.7 ± 5.1°	11.9°	30.8%	0.765
	CV	19.6 ± 9.6%	5.7 ± 5.8%	18.8%	330.0%	0.479
	asym.	18.2 ± 9.5%	7.2 ± 5.4%	16.6%	232.5%	0.459

Abbreviations: dsupp time: double support time; FPA: foot progression angle; ROM: range of motion; CV: coefficient of variation; asym.: asymmetry; RMSE: absolute and relative root mean square error; ICC: intraclass correlation coefficient.

## Data Availability

Sample datasets and gait analysis scripts used in this study are publicly available at https://github.com/DSGZ-MotionLab/vGait, accessed on 19 December 2024. The complete data from this study can be obtained upon reasonable request from M.W. The participants did not consent to the publication of their sensor data in open repositories in accordance with European data protection laws.
